# Case-Control Pilot Study on Acute Diarrheal Disease in a Geographically Defined Pediatric Population in a Middle Income Country

**DOI:** 10.1155/2017/6357597

**Published:** 2017-08-10

**Authors:** Ana E. Farfán-García, Chengxian Zhang, Aamer Imdad, Monica Y. Arias-Guerrero, Nayibe T. Sánchez-Alvarez, Rikhil Shah, Junaid Iqbal, Maria E. Tamborski, Oscar G. Gómez-Duarte

**Affiliations:** ^1^Grupo de Investigación en Manejo Clínico (CliniUDES), Programa de Bacteriología y Laboratorio Clínico, Facultad de Ciencias de la Salud, Universidad de Santander (UDES), Bucaramanga, Colombia; ^2^Division of Pediatric Infectious Diseases, Department of Pediatrics, Vanderbilt University School of Medicine, Nashville, TN, USA; ^3^Division of Pediatric Gastroenterology, Hepatology, and Nutrition, Department of Pediatrics, Vanderbilt University School of Medicine, Nashville, TN, USA

## Abstract

**Introduction:**

Acute diarrheal disease (ADD) is a common cause of morbidity and mortality in children under 5 years of age. Understanding of the etiology of ADD is lacking in most low and middle income countries because reference laboratories detect* limited number of pathogens*. The objective of this study was to determine the feasibility to conduct a comprehensive case-control study to survey diarrheal pathogens among children with and without moderate-to-severe ADD.

**Materials and Methods:**

Microbiology and molecular-based techniques were used to detect viral, bacterial, and parasitic enteropathogens. The study was conducted in Bucaramanga, Colombia, after Institutional Review Board approval was obtained.

**Results:**

Ninety children less than 5 years of age were recruited after a written informed consent was obtained from parents or guardians. Forty-five subjects served as cases with ADD and 45 as controls. Thirty-six subjects out of 90 (40.0%) were positive for at least one enteropathogen, that is, 20 (44.4%) cases and 16 (35.5%) controls.

**Conclusions:**

The three most common enteric pathogens were enteroaggregative* E. coli* (10.0%),* Norovirus* (6.7%), and* Salmonella *spp. (5.6%). The* E. coli *pathogens were 18.8% of all infections making them the most frequent pathogens. Half of ADD cases were negative for any pathogens.

## 1. Introduction

Acute diarrheal disease (ADD) is the most frequent cause of childhood illness and a leading cause of death in children less than 5 years of age [1]. In 2010, there were 1.731 billion episodes of diarrhea that lead to 700,000 deaths globally, mainly in low and middle income countries [2]. Most episodes of ADD are relatively mild to moderate; however about 2% of cases lead to severe diarrhea that is a risk factor for significant morbidity and mortality [2]. In addition to the risk of mortality from severe cases, recurrent episodes of ADD increase the risk of malnutrition. A recent study showed that odds of stunting increase by 1.13 times for every 5 episodes of diarrhea in children less 2 years of age [3]. Stunting is an important nutritional indicator as it represents long-term nutritional deficiency and is associated with cognitive deficits [2, 4]. In rare cases, selective enteropathogens like* E. coli* and* Campylobacter* can lead to severe morbidities (irrespective of severity of diarrhea), like hemolytic uremic syndrome and Guillain-Barre syndrome, among other conditions [5, 6].

The etiology and incidence of ADD vary in different regions of the world. The “Africa” and “Americas” regions have the highest incidence of ADD (about 3.3 and 3.2 episodes per child year, resp.), while Southeast Asia and Africa have the highest burden of severe ADD and diarrhea-related mortality [2, 7]. Infectious agents including viruses, bacteria, and parasites can cause ADD and their prevalence is reported to be different in different regions of the world [8]. Colombia has an estimated incidence of ADD in the range of 3.12 to 6.03 per child year with the highest incidence in infants 6–11 months of age [7]. The etiology of ADD in Colombia had been reported in a few studies; however, most of the studies were small and did not aim to look for all the etiologic agents (e.g., those caused by* E. coli* pathotypes and caliciviruses). Moreover, the microbiologic detection methods had limited sensitivity [9–12]. The objective of the current study was to determine the feasibility to conduct a large, matched, case-control study to examine the association of viral, bacterial, and parasitic enteropathogens with ADD in children of 2 weeks to 5 years of age in Colombia using advanced microbiologic and molecular techniques. Our work describes the methodology used in the study and results from pilot data. Such detailed studies, aimed at understanding the etiology of ADD, are important to guide appropriate future treatment and public health preventative interventions. 

## 2. Materials and Methods

### 2.1. Study Site

This study was conducted in the Bucaramanga metropolitan area, Colombia, from August to December 2013. The metropolitan area includes four municipalities: Bucaramanga, Girón, Piedecuesta, and Floridablanca. This city has a population of about 525,000 and is located on a plateau of the Andes at 959 meters above sea level, with an average temperature of 23°C and annual rainfall of 1041 mm. The city has over 90% coverage for basic utilities including those for water, electricity, telephone, gas, and garbage collection with a frequency of three times a week [13]. The coverage of pediatric hospital beds in this region is 0.48 per 1000 population for children under 12 years of age. In 2010, the under-5 mortality rate in Colombia was 5.26 per 100,000 children, decreasing from 10.68 in 2005. ADD is the third leading cause of morbidity among children less than 5 years of age in Santander, Colombia [14]. The study was conducted in five outpatient and inpatient pediatric facilities of four major medical institutions: Unidad Intermedia Materno Infantil Santa Teresita (UIMIST), Centro de Salud el Rosario, Fundación Oftalmológica de Santander Carlos Ardila Lulle (FOSCAL), Hospital Local del Norte, Clínica Materno Infantil San Luis, and Hospital San Juan de Dios de Floridablanca.

### 2.2. Study Design

This was a prospective, matched-for-age, case-control study to determine the etiology of ADD in children of 2 weeks to 59 months of age in the Bucaramanga metropolitan area, Colombia. This study was approved by Institutional Review Board, Vanderbilt University School of Medicine (IRB number 130327). Informed consent for subject participation was obtained from parents or guardians as all participants were children less than 5 years of age.  Table 1 gives inclusion/exclusion criteria for cases and controls. Patients were recruited from emergency, inpatient, and outpatient facilities of the above-mentioned medical centers. Controls were recruited for matching for age and treatment facility. After informed written consent was obtained, an interview questionnaire was administered to the subject's parents or guardians at enrollment and at 2 and 6 weeks after that. The interview was conducted and recorded in Spanish, by trained study clinical researchers. Information initially captured by interviewers in paper-based questionnaires was digitized and stored in the REDCap (Research Electronic Data Capture; https://www.project-redcap.org) database. REDCap is a secure, web-based application designed to capture data for research studies, providing an intuitive interface for validated data entry, audit trails for tracking data manipulation and export procedures for seamless data downloads to common statistical packages, and procedures for importing data from external sources [15]. Data collected from subjects included demographics, medical history, epidemiological factors, socioeconomic factors, nutrition, education, immunization history, water sources, and housing. For cases, information was obtained about clinical manifestations and specifically about characteristics of diarrhea, vomiting, abdominal pain, and dehydration.

### 2.3. Stool Samples Collection and Processing

**Figure 1:**
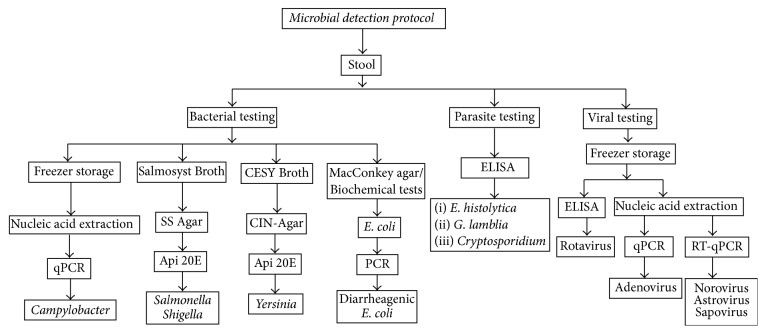
Microbial detection methods to identify bacterial, viral, and parasitic agents from stools samples of study subjects. PCR for diarrheagenic* E. coli* was to detect enterotoxigenic, enteropathogenic, Shiga-toxin producing, diffusely adherent, and enteroinvasive* E. coli* as well as emergent* E. coli* pathotypes.

Stools samples were collected for detection of viral, bacterial, and parasitic intestinal pathogens. Stool collection occurred on the day of enrollment or up to 1 week after that. Samples were collected spontaneously from subjects in disposable plastic bottles with wide-mouths and were transported within first four hours to the Laboratory of Biomedical and Biotechnological Research (LBBR) at the Universidad de Santander, Bucaramanga, Colombia. To ensure recovery of bacteria isolates, Cary Blair (Copan Transystem®, Brescia, Italy) transport media were used at the time of sample collection. Initially, the stool samples were examined for consistency, color, presence of mucus, and blood. Aliquots of stool specimens were made and divided into those to be tested immediately and those for storage at −20°C and at −80°C.  Figure 1 shows the microbial detection protocols of the stool samples.

### 2.4. Bacterial Processing and Testing

Bacteriological differential media were used to screen for diarrheagenic bacteria including* Salmonella *spp.,* Shigella *spp.,* Yersinia *spp., and* E. coli*. To screen and further identify* E. coli*, stool samples were initially plated on MacConkey (Merck®, Darmstadt, Germany) agar. Five individual lactose fermenter isolates that had metallic shine on Eosin Methylene Blue (EMB) agar (Merck, Darmstadt, Germany) were subsequently processed with biochemical assays for* E. coli* confirmation. Salmosyst Broth (Merck, Darmstadt, Germany) with supplements (Merck, Darmstadt, Germany) was used for isolation of* Shigella* and* Salmonella* species and CESY Broth (Merck, Darmstadt, Germany) was used for recovery of* Yersinia* at 37°C for 24 hours, respectively (Figure 1). Bacteria able to grow in selective media were subsequently cultured in either* Salmonella*-*Shigella* (SS) agar (Scharlau®, Sentmenat, Spain) or CIN-Agar (Merck, Darmstadt, Germany) for further selection of* Salmonella*,* Shigella,* or* Yersinia* species.

### 2.5. Detection of Bacterial Pathogens

After preliminary identification of bacterial species, suspicious colonies were selected and subjected to biochemical tests.* Salmonella, Shigella,* and* Yersinia* were identified by API20E (bioMériux®, Marcy-I'Etoile, France). Potential* E. coli* colonies were also evaluated in SIM medium (Merck, Darmstadt, Germany).* E. coli* isolates, reported positive for mobility and indole, negative for sulfide production, and positive for* uidA* DNA amplification by PCR (Supplementary Table 1, in Supplementary Material available online at https://doi.org/10.1155/2017/6357597), were confirmed as* E. coli*.* E. coli *confirmation was conducted randomly with API20E testing on a fraction of identified* E. coli*. All bacterial isolates were stored at −80°C in Luria Broth (LB) (Scharlau, Sentmenat, Spain) supplemented with 15% glycerol (Merck, Darmstadt, Germany).

The detection of* Campylobacter* in the pilot study was conducted by bacteria isolation and biochemical testing described above and quantitative PCR (qPCR) described previously [16]. Briefly, human stool samples were 1 : 10 diluted in DEPC-treated water (Life Technologies, Carlsbad, USA), vortexed, and centrifuged. DNA extraction was performed using a QIAamp® DNA stool mini kit (Qiagen, Valencia, CA). DNA was stored at −20°C until use. A qPCR assay was developed using a previously described assay on CFX96 Touch™ Real-Time PCR Detection System (Bio-Rad, Hercules, California, USA) for* C. jejuni/C. coli* [17]. Each well included a 25 *µ*l reaction mixture with 1 *µ*l of DNA sample, 12.5 *µ*l of TaqMan® environmental master mix, 9 *µ*l of nuclease free water, 1 *µ*l of each primer of cadF-F and cadF-R at final concentration of 0.4 *µ*M, and 0.5 *µ*l of cadF-P at final concentration of 0.2 *µ*M (Supplementary Table 2). The cycling conditions were as follows: 95°C for 10 min, followed by 45 cycles of 95°C for 15 s and 55°C for 1 min.

### 2.6. Detection of Diarrheagenic* Escherichia coli* by DNA Amplification

Bacterial stocks of clinical* E. coli *isolates were inoculated on LB plate (Scharlau, Sentmenat, Spain) and cultured at 37°C overnight. Cultures were harvested, suspended in one-milliliter sterile water, vortexed for 10 seconds, and centrifuged at maximum speed for two minutes. The supernatants that contained bacterial DNA were incubated for five minutes at 95°C on a thermocycler and used as DNA template in multiplex polymerase-chain reaction (mPCR). The mPCR was designed to detect six well-established pathogenic* E. coli* pathotypes including diffusely adherent* E. coli* (DAEC), enteroaggregative* E. coli* (EAEC), enteroinvasive* E. coli* (EIEC), enteropathogenic* E. coli* (EPEC), enterotoxigenic* E. coli* (ETEC), and Shiga-toxin producing* E. coli* (STEC), and we divided the mPCR into four reaction mixes with primers targeting specific virulence genes of each pathotype (Supplementary Table 1). The strains used as controls in the mPCR included the following: negative control DH5*α E. coli*, negative control TOP10F'* E. coli*, C1845 DAEC, JM221 EAEC, EC-12 EIEC, E2348/69 EPEC, E9034A ETEC, and 2060-004 EHEC [18]. Reaction mix 1 targets* lngA*,* bfpA*, and* stx2* genes; reaction mix 2 targets* eae*,* daaE*,* ipaD*, and* aaiC* genes; reaction mix 3 targets* ipaH*,* escN*,* aggR*, and* estA* genes; reaction mix 4 targets* elt* and* stx1* genes. In each 25 *µ*l mPCR reaction, we add 21 *µ*l Platinum® Blue PCR SuperMix (Life Technologies, Carlsbad, USA), 2 *µ*l DNA template, and 2 *µ*l primer mixes specifically indicated above. The final concentration of each primer in mPCR reaction was 0.4 *µ*M. The thermocycling was set as 94°C for 5 minutes, 40 cycles of denaturing at 94°C for 30 seconds, annealing at 58°C for 30 seconds, and extension at 72°C for 60 seconds, and final extension at 72°C for 5 minutes. The annealing temperature for reaction 4 was set at 60°C to increase the PCR specificity. The PCR products were electrophoresed on 2.0% agarose gel containing SYBR safe DNA gel stain (Life Technologies, Carlsbad, USA) and visualized under UV exposure.

### 2.7. Detection of Viral Pathogens

RNA extracted from human stool samples were used for detection of* Norovirus*, astrovirus, and* Sapovirus*. Briefly, human stool samples were 1 : 10 diluted in DEPC-treated water (Life Technologies, Carlsbad, USA), vortexed, and centrifuged. The supernatants were mixed with internal RNA virus control MS2 phage and the mixtures were processed for RNA extraction by QIAamp Viral RNA Mini Kit (QIAgen, Valencia, USA) following manufacturer's instructions. Detection of* Norovirus* GI and GII was conducted according to the US CDC protocol: GI/GII* Norovirus* Multiplex (TaqMan Life Technologies, Carlsbad, USA) real-time PCR (RT-PCR) Assay [19]. The reverse-transcription quantitative PCR (RT-qPCR) was conducted using Ag-Path One-Step RT-PCR Kit (Life Technologies, Carlsbad, USA) on CFX96 Touch Real-Time PCR Detection System (Bio-Rad, Hercules, California, USA). The 22 *µ*l master mix was prepared (Supplementary Table 2) and loaded on PCR plates and mixed with 3 *µ*l viral RNA preparation as template. RNA preparations negative and positive for* Norovirus* GI and GII were obtained from deidentified human stool samples in our laboratory collection and used as controls. In addition, water as a nontemplate control (NTC) was also included in the RT-QPCR assay. The thermocycling was set as 45°C for 10 minutes, 95°C for 10 minutes, 40 cycles of 95°C for 15 seconds, and 60°C for 1 minute. The* Norovirus* GI, GII, and MS2 phage were detected at detection channels of FAM, Cy5, and HEX. The threshold was set on the midpoint of the linear position of the curve and a sample was considered positive when a curve crosses the threshold before or at a Ct of 40.

Astrovirus and* Sapovirus* detection were conducted from isolated RNA (see above) according to the United States Center for Disease Control (CDC) protocol: Astrovirus/Sapovirus Duplex Real-Time (TaqMan) RT-QPCR Assay [20]. The duplex RT-qPCR was conducted using Ag-Path One-Step RT-PCR Kit (Life Technologies, Carlsbad, USA) on CFX96 Touch Real-Time PCR Detection System (Bio-Rad, Hercules, California, USA). The 22 *µ*l master mix was prepared according to Supplementary Table 2 and added to PCR plates followed by addition of 3 *µ*l viral RNA preparation as template. RNA preparation from astrovirus and* Sapovirus* negative and positive human stool samples were negative and positive controls in qRT-PCR, and water was nontemplate control (NTC). The thermocycling was set as 45°C for 10 minutes, 95°C for 10 minutes, 40 cycles of 95°C for 15 seconds, and 60°C for 1 minute. The astrovirus and* Sapovirus* were detected at detection channels of HEX and FAM. The threshold was set on the midpoint of the linear position of the curve and a sample was considered positive when a curve crosses the threshold before or at a Ct of 40.

Adenovirus detection from DNA isolated from stool samples was performed by qPCR as described previously [21]. The DNA isolation protocol from stools has been described above in section describing detection of bacterial pathogens. The qPCR was conducted using Ag-Path One-Step RT-PCR Kit (Life Technologies, Carlsbad, USA) on CFX96 Touch Real-Time PCR Detection System (Bio-Rad, Hercules, California, USA). The 22 *µ*l master mix was prepared according to Supplementary Table 2 and added to PCR plates followed by addition of 3 *µ*l DNA preparation as template. DNA samples obtained from adenovirus negative and positive human stool samples were used as negative and positive controls, respectively. Water was used as a nontemplate control (NTC). The thermocycling was set as 45°C for 10 minutes, 95°C for 10 minutes, 40 cycles of 95°C for 15 seconds, and 60°C for 1 minute. The adenovirus was detected at detection channels of FAM. The threshold was set on the midpoint of the linear position of the curve and a sample was considered positive when a curve crosses the threshold before or at a Ct of 40.

Identification of* Rotavirus* was done by the* Rotavirus* Stool Antigen Detection ELISA assay according to manufacturer's instructions (Diagnostic Automation®, Calabasas, USA). Briefly, the stool samples were diluted 1 : 5 in diluted wash buffer. 96-well microtitre plates containing polyclonal anti-*Rotavirus* antibodies were used. After 100 *µ*l of each supernatant was added to each well including negative and positive control and incubated at room temperature for 30 minutes and then washed three times, anti-*Rotavirus* monoclonal antibodies with Thimerosal were added. This was followed by addition of anti-mouse antibodies conjugated to horseradish peroxidase with Thimerosal. These steps were followed by 5 minutes' incubation at room temperature and washed 3 times with 300 *µ*l of wash buffer and 50 *μ*l volume of each reagent was used. After that, tetramethylbenzidine (TMB) and peroxide mixture was added and allowed to react for 5 minutes. Finally, reaction was stopped by adding 5% phosphoric acid solution. The optical density (OD) of the wells was read at 450 nm wavelength with 620 nm reference filter in an ELISA reader (Bio-Rad, USA). OD value ≥ 0.15 was considered as positive.

All stool samples were processed for detection of* Giardia lamblia*,* Entamoeba histolytica*/*E. dispar,* and* Cryptosporidium* antigens by direct ELISA (Diagnostic Automation, Calabasas, USA) following manufacturer's instructions.

### 2.8. Statistical Analysis

Data for basic demographics were described as means, medians, and percentages. To estimate the association of a particular pathogen with ADD, odds ratios (OR) were calculated with 95% confidence interval. These results were described as crude analyses for pilot data; however, for the future, larger main study, OR would be adjusted by multiregression analyses for potential confounders like age, location, coinfection, water source, nutritional status, socioeconomic status, breastfeeding, and formula feeding, among other variables. Results of association of a pathogen with ADD were stratified according to age, that is, 2 weeks to 11 months, 12–23 mo, and 24–59 months. Proportions were compared by Chi-square analysis or Fisher's Exact-Boschloo test (confidence interval method with confidence coefficient 0.999). Statistical significance was set as a *P* value < 0.05. All statistical analysis was performed using STATA version 13.

## 3. Results

### 3.1. Characteristics of Cases and Controls

**Figure 2:**
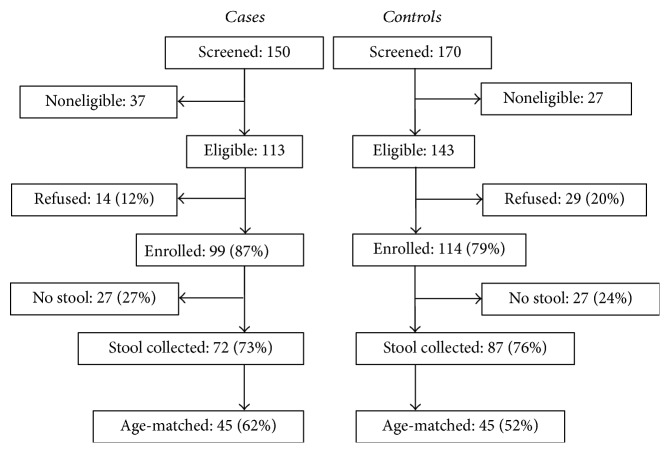
Enrollment of cases and controls for the childhood diarrhea study in Bucaramanga, Colombia.

This pilot case-control study was conducted on 90 subjects, 45 cases and 45 controls.  Figure 2 gives the details of enrollment.  Table 2 gives details of basic demographics. Forty-three males and 47 females were enrolled in the pilot study, and the difference was not statistically significant (*P* = 0.67). The age of the subjects ranged from 0 to 55 months with the mean age at 17.7 months and the median age at 12 months. The majority of subjects were in age ranged 2 weeks to <12 months (*n* = 42, 46.6%). The proportions of subjects aged 12 to <24 and 24 to <60 months were 22.2% and 31.1%, respectively. Mestizo was the most prevalent racial group with 86 (95.6%) subjects out of 95, followed by White with 4 (4.4%) (Table 2).

### 3.2. Infections among Cases and Controls

There were 36 subjects out of 90 (40.0%) with at least one enteropathogen identified in stools, that is, 20 (44.4%) cases and 16 (35.5%) controls (Table 3). From a total of 7 coinfections among subjects, 6 (13.3%) occurred among cases and 1 (2.2%) in a control (Table 3). Twenty-five cases were negative for any pathogens indicating that no infectious etiology was detected in 55.5% of cases.

### 3.3. Bacterial Pathogens Detected among Cases and Controls

The total number of* E. coli* intestinal pathogens was 17 (18.8%) (Table 4), 13 of them corresponded to single infections and 4 of them to coinfections.* E. coli* pathogens were the most frequent diarrheagenic pathogen group in this pediatric population. EAEC was the most frequent* E. coli* pathogen with a total of 9 (10%) infections, 6 (13.3%) among cases and 3 (6.7%) in controls (Table 4). Other diarrheagenic* E. coli* identified included EPEC, DAEC, ETEC, and EIEC. All EPEC isolates identified were atypical EPEC. No STEC were detected in this population. EIEC and DAEC were only detected among cases (Table 4).

The only non-*E. coli* bacterial pathogen detected in this pilot study was* Salmonella*. A total of 5 (5.6%) subjects were infected (Table 4), 3 of them were single infection and 2 coinfections. No* Campylobacter* spp.,* Shigella* spp., or* Yersinia* spp. were identified.* V. cholerae* was not investigated as Colombia is no longer endemic for these organisms. Overall, the total number of bacterial infections detected was 22, 15 among cases and 7 among controls.

### 3.4. Viral and Parasitic Pathogens Detected among Cases and Controls

A total of 14 (15.5%) viral infections were detected, 6 corresponded to single infections and 8 to coinfections (Table 4).* Norovirus* was identified in 6 subjects (6.6%), 3 were single infections and 3 coinfections.* Norovirus* was the most frequent viral infection (Table 4). Other viral infections detected included* Sapovirus* in 3 subjects (3.3%), adenovirus in 2 (2.2%) subjects,* Rotavirus* in 2 subjects (2.2%), and astrovirus in 1 subject (1.1%) (Table 4). The two diarrheagenic intestinal parasites identified were* Entamoeba histolytica/E. dispar* and* Giardia lamblia.* Out of 4 (4.4%) total* E. histolytica/E. dispar* infections, 2 were single infections and 2 were coinfections (Table 4). Out of 4 (4.4%) total* G. lamblia* infections, 1 was a single infection and 3 were coinfections.* Cryptosporidium *was not identified.

Coinfections were detected in a total of 7 (10%) subjects, 6 cases and 1 control (Table 3). Detected coinfections included the following: EAEC +* Norovirus*, EAEC +* Rotavirus*, EIEC +* Norovirus*,* E. histolytica/E. dispar* + EAEC +* Norovirus* GI,* Salmonella* +* Rotavirus*,* Salmonella* +* E. histolytica *+* E. dispar, *and* Giardia lamblia* +* Sapovirus*.

### 3.5. Socioeconomic Risk Factors among Cases and Healthy Controls

Both cases and controls were members of lower socioeconomic levels based on household income and housing level. Most parents or guardians had some level of education, and 95.5% of cases and controls were insured (Supplementary Table 3). No significant differences were identified between cases and controls with respect to socioeconomic factors, animal exposure, AGE contacts, or daycare attendance (Supplementary Tables 3 and 4). All households had access to tap water, sewage system, and garbage collection, except 1 case without access to the sewage system (Supplementary Table 4).

## 4. Discussion

This pilot case-control study reports the objectives, methodology, and preliminary results of a larger case-control study on AGE that is targeted to include approximately 450 cases and 450 controls. Coordinated efforts from members of local medical centers, research personnel, and community in general made a successful completion of this case-control pilot study possible. The preliminary results showed that 44% of children with ADD were positive for enteropathogens. Interestingly, 35% of healthy controls were also positive for enteropathogens.* E. coli* enteropathogens were the most prevalent organisms in the study population and higher numbers of cases of ADD had a coinfection with other organisms compared to controls.

This study applied comprehensive microbiological and molecular methods to detect viruses, bacteria, and parasites in the study population, which is expected to increase the sensitivity of enteropathogen detection. The controls were age-matched and also selected from the same study location from where the cases were recruited. This is important as differences in location of recruitment of cases and controls can lead to potential environmental difference that can place one or the other group at a higher risk of particular infectious agent. Cases were selected from outpatient, inpatient, and emergency department settings and are expected to represent the spectrum of severity of ADD. We found that bacteria were the most common type of pathogens associated with ADD. This is in contrast with other studies from Colombia reporting viruses, including* Rotavirus*, as the most common pathogens [22, 23]. One explanation for the low* Rotavirus* incidence among ADD cases may be the introduction of* Rotavirus* vaccine in Colombia in 2009 that might have contributed to a decline in* Rotavirus* infections and disease severity [24, 25]. In addition, our study enrolled ADD cases with moderate-to-severe diarrhea. Since* Rotavirus* vaccines protect not only against* Rotavirus* infection but also against severe disease, it is highly likely that the number of cases with mild diarrhea secondary to* Rotavirus* may have been excluded.

In this study, 35.5% of the control subjects were identified as enteropathogen-positive. This information is relevant because Colombia, a middle income, is expected to have lower risk factors for infection compared to low income countries, yet our results show high proportion of infections in controls as previously reported by case-control studies conducted in low income countries [8]. The reasons that people without ADD excrete pathogens have been well described previously and thought to be attributable to characteristics of pathogens and host factors [26]. One example of that is the carriage state of* Salmonella typhi*, which results in persistent shedding for months to years in asymptomatic individuals [27]. Carriage state has also been reported for other organisms including* Campylobacter jejuni* [28, 29],* Norovirus* GI and GII [30], and* Shigella* spp. [31]. Due to heterogeneity of pathogenicity among pathogens, some strains appear to be more prone to cause clinical disease than others [26]. For instance, EPEC strains E2348/69 and E851/71 caused higher attack rates and more severe ADD, whereas EPEC strain E74/68 elicited lower attack rates or milder diarrheal diseases or no illness at all [26, 31, 32]. Thus, control subjects may appear healthy when they are asymptomatically infected with less virulent strains.

The Global Enteric Multicenter Study (GEMS) is the largest, multicenter study conducted to date to assess the etiology and epidemiology of moderate-to-severe diarrhea in children less than 5 years of age [8]. This study was conducted in 5 different countries, 4 from Africa and 3 from Southeast Asia. The study demonstrated that four enteropathogens were the most common cause of diarrhea in children and these include* Rotavirus*,* Cryptosporidium*, enterotoxigenic* Escherichia coli* producing heat stable toxin, and* Shigella*. This study also demonstrated that there was great variability of enteropathogens in different study sites and some of the pathogens were commoner at one place compared to other sites. The American continent region has the second highest incidence of ADD; however no comprehensive study has been done in this region to look at the etiology of ADD [2]. Most of the previous studies conducted in this region had small sample size [9, 25, 33–36] and also did not use advance microbiological techniques to detect pathogens. This aspect is important in two ways, one is that newer methods have better sensitivities [18], and, second, the spectrum of ADD-causing agents might be changing after the introduction of* Rotavirus* vaccine. We hope to fill the gaps in knowledge about the etiology of ADD in this region. Knowledge of etiology of ADD is important as it can help define targets for prevention such as vaccines and sometime leads to identification of source of infection; for example, certain pathogenic* E. coli* can be transmitted via contaminated food [37].

The main objective of this paper was to evaluate feasibility to conduct a larger study. We were able to conduct successfully this pilot study, the detailed methodology was performed both on site and in the USA, and data was collected for statistical analysis. This information provided us with the design of a larger study in terms of logistics and sample size determination. Based on pilot data, we anticipate that bacterial and viral etiologies may be the most common cause of ADD in this study population with pathogenic* E. coli *being possibly more prevalent. Preliminary data also identified an emergent* E. coli* pathogen in a case with ADD, identified as an enteroinvasive* E. coli*. This emergent EIEC pathotype has a strong biofilm phenotype not previously reported among EIEC clinical isolates (our unpublished results).

There were limitations in this study. The small sample size of this pilot study prevented us from conducting advanced analyses. Cases were selected from patients coming to a medical facility and would not include the cases in which child was not brought to the hospital or was taken to other hospitals that were not part of this study. Pilot data were collected from August to December only; therefore data are not representative of time periods. Up to 55.6% of all ADD cases were negative for any pathogen tested, and this may be explained by chance, taking into account the low number of subjects in this pilot study. An alternative explanation is that our study used less sensitive, yet more specific, assays including conventional microbiology for the detection of all bacterial pathogens, and ELISA assays for* Rotavirus*,* Cryptosporidium*,* G. lamblia*, and* E. histolytica*. We aim to overcome some of these limitations in a larger study.

## 5. Conclusion

This pilot case-control study showed that children with ADD and healthy controls had high proportion of enteropathogens detected in stools, suggesting a high level of healthy carriage state within this population. More than 50% of the cases of ADD were negative for any enteropathogen, indicating that in half of the cases the cause of ADD remains unknown. Bacteria were commoner than viruses and most common organisms were* E. coli* pathotypes. The proportion of coinfections among cases of ADD was higher than in controls suggesting that coinfection, rather than single infections, may contribute to ADD in this population.

## Supplementary Material

Supplementary Table 1. Primer and probes for the conventional multiplex PCR and real-time RT-PCR.
Supplementary Table 2. Reaction mix for norovirus, astrovirus, *Sapovirus*, and *Campylobacter* PCR and reaction mix for GI/GII Norovirus Multiplex RT-qPCR Assay.
Supplementary Table 3. Socioeconomic risk factors associated with acute diarrheal disease in cases and healthy controls.
Supplementary Table 4. Epidemiological risk factors associated with acute diarrheal disease in cases and healthy controls.

## Figures and Tables

**Table 1:** Inclusion/exclusion criteriafor cases and controls.

Cases
*Inclusion criteria:*
Children less than 5 years old
Presence of acute, moderate-to-severe diarrhea and/or vomiting within the past 10 days. The World Health Organization defines diarrhea as 3 or more episodes of loose or liquid stools
within 24 hours
Children who are not part of this study as a case (not previously recorded to have diarrhea within the last 60 days)
Diarrhea is moderate to severe and must meet at least one of the following criteria:
(i) Sunken eyes, confirmed by parent/caretaker
(ii) Loss of skin turgor by skin pinch (≤2 s slow or >2 very slow)
(iii) Intravenous rehydration prescribed or administered
(iv) Dysentery (1 or more bloody stools)
(v) Evaluated in the emergency department or admitted to the hospital for diarrhea
Children will still be eligible for enrollment even if they have received antibiotics within the last 14 days
*Exclusion criteria:*
Children older than 60 months of age
Children who reside outside of the metropolitan area of Bucaramanga, Colombia
Presence of chronic diarrhea (>10 days) or other comorbid conditions such as Crohn's disease or ulcerative colitis

Controls
*Inclusion criteria:*
Children less than 5 years old
Child who resides within the metropolitan area of Bucaramanga, Colombia
Absence of diarrhea or vomiting within the past 10 days
Matched to cases for age. Age matching is ±2 months for 0–11 months, ±4 months for 12–59 months (can not exceed the stratum boundaries of the case)
*Exclusion criteria: *
Children older than 60 months of age
Child who does not reside within the Metropolitan area of Bucaramanga, Colombia
Presence of acute diarrhea, as defined by the WHO, in the previous 7 days (regardless of whether they develop diarrhea after enrollment)
Presence of chronic diarrhea (>10 days) or other comorbid conditions such as Crohn's disease or ulcerative colitis

**Table 2:** Sociodemographics of subjects.

	Number (%)		
Variable	Cases	Controls	*P*
*Gender*			
Male	20 (44.4)	23 (51.1)	0.67
Female	25 (55.6)	22 (48.9)	
*Race*			
Mestizo	42 (93.3)	44 (97.8)	0.62
White	3 (6.7)	1 (2.2)	
*Age group*			
0 to <12	21 (46.7)	21 (46.7)	1
12 to <24	10 (22.2)	10 (22.2)	1
24 to <60	14 (31.1)	14 (31.1)	1

**Table 3:** Proportion of infected subjects by either single or multiple pathogen (coinfections) in a case-control study.

Pathogens	Total		Cases		Controls		*P*
	*n*	%	*n*	%	*n*	%	
EAEC	6	6.7	3	6.7	3	6.7	1
DAEC	2	2.2	2	4.4	0	0.0	0.49
EPEC	3	3.3	1	2.2	2	4.4	1
EIEC	0	0.0	0	0.0	0	0.0	1
ETEC	2	2.2	1	2.2	1	2.2	1
*Salmonella*	3	3.3	2	4.4	1	2.2	1
*Rotavirus*	0	0.0	0	0.0	0	0.0	1
Adenovirus	2	2.2	2	4.4	0	0.0	0.494
Astrovirus	1	1.1	0	0.0	1	2.2	1
*Sapovirus*	2	2.2	1	2.2	1	2.2	1
*Norovirus*	3	3.3	0	0.0	3	6.7	0.241
*Entamoeba *spp.	2	2.2	1	2.2	1	2.2	1
*Giardia lamblia*	3	3.3	1	2.2	2	4.4	1
Coinfections	7	7.7	6	13.3	1	2.2	0.11
Negative	54	60	25	55.6	29	64.4	0.52
Total	90	100.0	45	100.0	45	100.0	1

EAEC = enteroaggregative *E. coli*, DAEC = diffusely adherent *E. coli*, EPEC = enteropathogenic* E. coli*, EIEC = enteroinvasive *E. coli*, ETEC = enterotoxigenic *E. coli, Entamoeba spp. = Entamoeba histolytica and Entamoeba dispar*; Fisher's Exact-Boschloo test was applied for analysis in this table.

**Table 4:** Proportion of individual pathogens detected among children with and without ADD.

Pathogens	Total		Cases		Controls		*P*
	*n*	%	*n*	%	*n*	%	
EAEC	9	10.0	6	13.3	3	6.7	0.4845
DAEC	2	2.2	2	4.4	0	0.0	0.4944
EPEC	3	3.3	1	2.2	2	4.4	1
EIEC	1	1.1	1	2.2	0	0.0	1
ETEC	2	2.2	1	2.2	1	2.2	1
*Salmonella*	5	5.6	4	8.9	1	2.2	0.36
Rotavirus	2	2.2	2	4.4	0	0.0	0.4944
Adenovirus	2	2.2	2	4.4	0	0.0	0.4944
Astrovirus	1	1.1	0	0.0	1	2.2	1
Sapovirus	3	3.3	1	2.2	2	4.4	1
Norovirus	6	6.7	3	6.7	3	6.7	1
*Entamoeba *spp.	4	4.4	3	6.7	1	2.2	0.6164
*Giardia lamblia*	4	4.4	1	2.2	3	6.7	0.6164

EAEC = enteroaggregative *E. coli*; DAEC = diffusely adherent *E. coli*; EPEC = enteropathogenic* E. coli*; EIEC = enteroinvasive *E. coli*; ETEC = enterotoxigenic *E. coli*; *Entamoeba spp. = Entamoeba histolytica/Entamoeba dispar*; Fisher's Exact-Boschloo test was applied for analysis in this table.

## Additional Points

*Availability of Data and Materials*. Information initially captured by interviewers in paper-based questionnaires and microbiological information was digitized and stored in the REDCap (Research Electronic Data Capture; https://www.project-redcap.org) database. REDCap is a secure, web-based application designed to capture data for research studies, providing an intuitive interface for validated data entry, audit trails for tracking data manipulation and export procedures for seamless data downloads to common statistical packages, and procedures for importing data from external sources. This data is available upon request. This data is not publically available as this study is ongoing. The authors are only presenting data from a pilot study. The data and the analysis are however available upon request.
