# Supplementary material for: Case-Control Pilot Study on Acute Diarrheal Disease in a Geographically Defined Pediatric Population in a Middle Income Country
Source: Int J Pediatr. 2017 Aug 10;2017:6357597. doi: 10.1155/2017/6357597 (PMC5569627; doi:10.1155/2017/6357597)
Supplement: Supplementary file 1 — Supplementary Table 1. Primer and probes for the conventional multiplex PCR and real-time RT-PCR. Supplementary Table 2. Reaction mix for norovirus, astrovirus, Sapovirus, and Campylobacter PCR and reaction mix for GI/GII Norovirus Multiplex RT-qPCR Assay. Supplementary Table 3. Socioeconomic risk factors associated with acute diarrheal disease in cases and healthy controls. Supplementary Table 4. Epidemiological risk factors associated with acute diarrheal disease in cases and healthy controls. [file 6357597.f1.pdf]

## Supplementary Material

**STable 1.** Primer and probes for the conventional multiplex PCR and real-time RT-PCR

| Organisms | Primers | Target | Primer Sequences (5'-3') | Amplicons (bp) | References |
|---|---|---|---|---|---|
| DAEC | daaE-F | *daaE* | GAA CGT TGG TTA ATG TGG GGT AA | 542 | 38 |
| | daaE-R | | TAT TCA CCG GTC GGT TAT CAG T | | |
| EAEC | aaiC-F | *aaiC* | ATT GTC CTC AGG CAT TTC AC | 215 | 39 |
| | aaiC-R | | ACG ACA CCC CTG ATA AAC AA | | |
| | aggR-F | *aggR* | GTA TAC ACA AAA GAA GGA AGC | 254 | 40 |
| | aggR-R | | ACA GAA TCG TCA GCA TCA GC | | |
| EIEC | ipaH-F | *ipaH* | CTC GGC ACG TTT TAA TAG TCT GG | 933 | 38 |
| | ipaH-R | | GTG GAG AGC TGA AGT TTC TCT GC | | |
| EPEC | Bfp-F | *bfpA* | AAT GGT GCT TGC GCT TGC TGC | 326 | 40 |
| | Bfp-R | | GCC GCT TTA TCC AAC CTG GTA | | |
| | eae384-F | *eae* | GAC CCG GCA CAA GCA TAA GC | 384 | 39 |
| | eae384-R | | CCA CCT GCA GCA ACA AGA GG | | |
| | eae917-F | *eae* | CTG AAC GGC GAT TAC GCG AA | 917 | 40 |
| | eae917-R | | CGA GAC GAT ACG ATC CAG | | |
| ETEC | LT-F | *Elt* | CAC ACG GAG CTC CTC AGT C | 508 | 39 |
| | LT-R | | CCC CCA GCC TAG CTT AGT TT | | |
| | ST-F | *est* | GCT AAA CCA GTA G/AGG TCT TCA AAA | 147 | 39 |
| | ST-R | | CCC GGT ACA G/AGC AGG ATT ACA ACA | | |
| | LngA1-F | *lngA* | ATGAGCCTGCTGGAAGTTATCATT | 621 | This study |
| | LngA621-R | | TTAACGGCTACCTAAAGTAATTGA | | |
| STEC | eae917-F | *eae* | CTG AAC GGC GAT TAC GCG AA | 917 | 40 |
| | eae917-R | | CGA GAC GAT ACG ATC CAG | | |
| | eae384-F | *eae* | GAC CCG GCA CAA GCA TAA GC | 384 | 39 |
| | eae384-R | | CCA CCT GCA GCA ACA AGA GG | | |
| | stx1-F | *stx1* | ATA AAT CGC CAT TCG TTG ACT AC | 180 | 39 |
| | stx1-R | | AGA ACG CCC ACT GAG ATC ATC | | |
| | stx2-F | *stx2* | GGC ACT GTC TGA AAC TGC TCC | 255 | 39 |
| | stx2-R | | TCG CCA GTT ATC TGA CAT TCT G | | |
| *E. coli* | uidA-F | *uidA* | GCGTCTGTTGACTGGCAGGTGGTGG | 503 | 18 |
| | uidA-R | | GTTGCCCGCTTCGAAACCAATGCCT | | |
| *Campylobacter* | cadF-F | *cadF* | CTGCTAAACCATAGAAATAAAATTTCTCAC | | 16 |
| | cadF-R | | CTTTGAAGGTAATTTAGATATGGATAATCG | | |
| Norovirus GI | Cog 1F | ORF1–ORF2 junction (GI) | CGYTGGATGCGNTTYCATGA | | 41 |
| | Cog 1R | | CTTAGACGCCATCATCATTYAC | | |

| | | | | |
|---|---|---|---|---|
| Norovirus GII | Cog 2F | ORF1–ORF2 junction (GII) | CARGARBCNATGTTYAGRTGGATGAG | 41 |
| | Cog 2R | | TCGACGCCATCTTCATTCACA | |
| MS phage | MS2.F | 289–387 | TGGCACTACCCCTCTCCGTATTCACG | 19 |
| | MS2.R | | GTACGGGCGACCCCACGATGAC | |
| Astrovirus | AsFF | ORF1b | GGC CAG ACT CAC AGA AGA GCA | 20 |
| | AsFr | | GTC CTG TGA CAC CTT GTT TCC TGA | |
| Sapovirus | SaV124F | ORF1 | GAY CAS GCT CTC GCY ACC TAC | 42 |
| | SaV1F | | TTG GCC CTC GCC ACC TAC | |
| | SaV5F | | TTT GAA CAA GCT GTG GCA TGC TAC | 42 |
| | SaV1245R | | CCC TCC ATY TCA AAC ACT A | |
| Adenovirus | JTVFF | Hexon | AAC TTT CTC TCT TAA TAG ACG CC | 44 |
| | JTVFR | | AGG GGG CTA GAA AAC AAA A | |

| Organisms | Probes | Target | Probe Sequences (5'-3') | References |
|---|---|---|---|---|
| Campylobacter | cadFP | cadF | [HEX]-CATTTTGACGATTTTTGGCTTGA-[BHQ2] | 16 |
| Norovirus GI | Ring 1E | ORF1–ORF2 junction (GI) | [6-FAM]-AGATYGCGRTCYCCTGTCCA-[Tamra] | 41 |
| Norovirus GII | Ring 2 | ORF1–ORF2 junction (GII) | [CY5]-TGGGAGGGCGATCGCAATCT-[BHQ2] | 41 |
| MS phage | MS2.P probe | neucleotide 289–387 | [5-HEX]-CACATCGATAGATCAAGGTGCCTACAAGC-[BHQ1] | 19 |
| Astrovirus | AstZFb | ORF1b | [5-HEX]-CCA TCG CAT TTG GAG GGG AGG ACC AGC GA-[BHQ1] | 20 |
| Sapovirus | SaV124TP | ORF1 | [6-FAM]-CCR CCT ATR AAC CA-[MGB-NQF] | 42 |
| | SaV5TP | ORF1 | [6-FAM]-TGC CAC CAA TGT ACC A-[MGB-NQF] | 42 |
| Adenovirus | JTVFAP | Hexon | [6-FAM]-CGA AGA GTG CCC GTG TCA GC-[BHQ1] | 44 |

DAEC = Diffusely adherent *E. coli*; EAEC = Enteroaggregative *E. coli*; EIEC = Enteroinvasive *E. coli*; EPEC = Enteropathogenic *E. coli*; ETEC = Enterotoxigenic *E. coli;* and STEC = Shiga toxin-producing *E. coli*

**STable 2.** Reaction Mix for norovirus, astrovirus, sapovirus and Campylobacter PCR

Reaction Mix for GI/GII Norovirus Multiplex RT-qPCR Assay

| Component | Volume per reaction (µl) | Final concentration |
|---|---|---|
| 2X RT-PCR buffer* | 12.5 | 1X |
| Nuclease-free water* | 1.08 | n/a |
| Detection Enhancer* | 1.67 | n/a |
| Cog1F (10 µM) | 1 | 400 nM |
| Cog1R (10 µM) | 1 | 400 nM |
| Ring 1E (10 µM) | 0.5 | 200 nM |
| Cog2F (10 µM) | 1 | 400 nM |
| Cog2R (10 µM) | 1 | 400 nM |
| Ring 2 (10 µM) | 0.5 | 200 nM |
| MS2.F (10 µM) | 0.25 | 100 nM |
| MS2.R (10 µM) | 0.25 | 100 nM |
| MS2.P (10 µM) | 0.25 | 100 nM |
| 25X RT-PCR enzyme* | 1 | 1x |
| RNA sample | 3 | |

Reaction Mix Astrovirus/Sapovirus Duplex RT-qPCR Assay

| Component | Volume per reaction (µl) | Final concentration |
|---|---|---|
| 2X RT-PCR buffer* | 12.5 | 1X |
| Nuclease-free water* | 2.5 | n/a |
| AsFF (10 µM) | 0.6 | 250 nM |
| AsFR (10 µM) | 0.6 | 250 nM |
| AstZFB (10 µM) | 0.25 | 100 nM |
| SaV 124F (10 µM) | 1 | 400 nM |
| SaV 1F (10 µM) | 1 | 400 nM |
| SaV 5F (10 µM) | 1 | 400 nM |
| SaV 1245R (10 µM) | 1 | 400 nM |
| SaV124TP (10 µM) | 0.25 | 100 nM |
| SaV 5TP (10 µM) | 0.25 | 100 nM |
| 25X RT-PCR enzyme* | 1 | 1x |
| RNA sample | 3 | |

Reaction Mix for adenovirus RT-qPCR Assay

| Component | Volume per reaction (µl) | Final concentration |
|---|---|---|
| 2X RT-PCR buffer* | 12.5 | 1X |
| Nuclease-free water* | 4.33 | n/a |
| Detection Enhancer* | 1.67 | n/a |
| Cog1F (10 µM) | 1 | 400 nM |
| Cog1R (10 µM) | 1 | 400 nM |
| Ring 1E (10 µM) | 0.5 | 200 nM |
| 25X RT-PCR enzyme* | 1 | 1x |
| RNA sample | 3 | |

Reaction Mix for Campylobacter qPCR Assay

| Component | Volume per reaction (µl) | Final concentration |
|---|---|---|
| TaqMan Environmental Master Mix 2.0 | 12.5 | 1X |
| Nuclease-free water | 9 | n/a |
| cadF-F (10 µM) | 1 | 400 nM |
| cadF-R (10 µM) | 1 | 400 nM |
| cadF-P (10 µM) | 0.5 | 200 nM |
| DNA sample | 1 | |

* included in the Ag-Path Kit

**STable 3.** Socio-economic risk factors associated with acute diarrheal disease in cases and healthy controls

| Risk factors | Cases | | Controls | | |
|---|---|---|---|---|---|
| | No. | % | No. | % | *P* |
| **Annual income*** | | | | | |
| Unknown | 6 | 13.3 | 7 | 15.6 | 0.74 |
| ≤1 | 25 | 55.6 | 26 | 57.8 | 0.83 |
| 1-2 | 10 | 22.2 | 10 | 22.2 | 0.92 |
| 2-6 | 4 | 8.9 | 2 | 4.4 | 0.58 |
| **Housing Levels** | | | | | |
| Unknown | 1 | 2.2 | 4 | 8.9 | 0.36 |
| 1 | 13 | 28.9 | 19 | 42.2 | 0.17 |
| 2 | 12 | 26.7 | 13 | 28.9 | 0.94 |
| 3 | 15 | 33.3 | 8 | 17.8 | 0.14 |
| 4 | 4 | 8.9 | 1 | 2.2 | 0.36 |
| 5 | 0 | 0 | 0 | 0.0 | N.A. |
| **Health System Insurance** | | | | | |
| Contributive | 31 | 68.9 | 22 | 48.9 | 0.086 |
| Subsidized | 9 | 20.0 | 21 | 46.7 | 0.02 |
| Private | 3 | 6.7 | 0 | 0.0 | 0.24 |
| Non-insured | 1 | 2.2 | 2 | 4.4 | 1 |
| Unknown | 1 | 2.2 | 0 | 0.0 | 1 |
| **Education of caregivers** | | | | | |
| Unknown | 1 | 2.2 | 0 | 0.0 | |
| None | 0 | 0.0 | 2 | 4.4 | 0.4944 |
| Elementary | 9 | 20.0 | 10 | 22.2 | 0.85 |
| Secondary | 18 | 40.0 | 19 | 42.2 | 0.83 |
| Technological school | 11 | 24.4 | 10 | 22.2 | 0.76 |
| University | 6 | 13.3 | 4 | 8.9 | 0.74 |

*: Income level 1 = One or less than one minimum salary per household per month (Colombian pesos $589,500)
N.A.=not applicable

**STable 4.** Epidemiological risk factors associated with acute diarrheal disease in cases and healthy controls

| Risk factors | Cases | | Controls | | |
|---|---|---|---|---|---|
| | **No.** | **%** | **No.** | **%** | ***P*** |
| Animals exposure | 23 | 51.1 | 25 | 55.6 | 0.83 |
| AGE Contacts outside home | 2 | 4.4 | 2 | 4.4 | 1 |
| Water sources | 45 | 100.0 | 45 | 100.0 | 1 |
| Garbage collection | 45 | 100.0 | 45 | 100.0 | 1 |
| Sewerage | 44 | 97.8 | 45 | 100.0 | 1 |
